# A Rare Factor in the Etiology of Loffler’s Pneumonia: *Fasciola hepatica*

**DOI:** 10.1590/0037-8682-0201-2023

**Published:** 2023-07-24

**Authors:** Buğra Kerget, Ferhan Kerget, Mehmet Eren Tuna

**Affiliations:** 1 Ataturk University School of Medicine, Department of Pulmonary Diseases, Yakutiye, Erzurum, Turkey. Ataturk University School of Medicine Department of Pulmonary Diseases Yakutiye Erzurum Turkey; 2 Health Sciences University, Erzurum Regional Education and Research Hospital, Depertmant of Infection Diseases and Clinical Microbiology, Erzurum, Turkey. Health Sciences University Erzurum Regional Education and Research Hospital Depertmant of Infection Diseases and Clinical Microbiology Erzurum Turkey

A 37-year-old woman presented to our outpatient clinic with exertional dyspnea and a dry cough. Posteroanterior chest radiography showed bluntness in the left sinus and a consolidated area with air bronchograms in the upper zone of the left lung. Her eosinophil count was 6400/ µL. She was thought to have eosinophilic pneumonia. Thoracic computed tomography showed an irregular consolidated area with air bronchograms in the upper lobe of the left lung, minimal pleural effusions in the lower lobe of the left lung, and heterogeneous lesions in the liver (Figure 1). Thoracentesis cytology revealed mixed-type inflammatory cells rich in eosinophils (60% eosinophils, 30% neutrophils, 5% macrophages, and 5% lymphocytes). *Fasciola hepatica* eggs were observed in the choledochal fluid obtained using endoscopic retrograde cholangiopancreatography (ERCP). Loffler’s pneumonia due to *Fasciola hepatica* infection of the lung parenchyma was considered. Triclabendazole treatment for *Fasciola hepatica* was initiated. After initiation of treatment, the patient’s symptoms regressed, and radiological examination showed significant regression in consolidation within the first month (Figure 2)**.** An eosinophilic reaction without direct parasitic involvement, accompanied by extrahepatic symptoms and signs is known as atypical fascioliasis^1^^,^^2^. Enzyme-linked immunosorbent assay has largely replaced fecal egg and parasite testing because of itss rapid turnaround time, high sensitivity, and ease of quantification. Adult parasites are occasionally detected during ERCP in patients with biliary obstruction, as observed in our case. Triclabendazole is effective against all stages of fascioliasis, with a cure rate of over 90%^3^.

**FIGURE 1:**
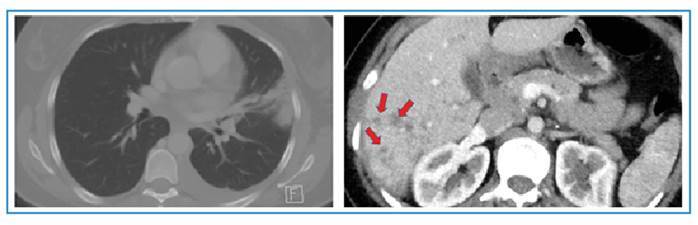
Thoracic computed tomography images showing a consolidated area with air bronchograms in the upper lobe of the left lung, minimal pleural effusions in the lower lobe of the left lung and heterogeneous lesions in the liver (red arrows).

**FIGURE 2:**
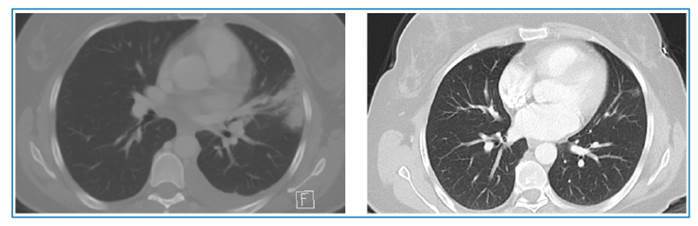
Thoraciccomputed tomography images taken one month later showing complete regression of the lesions in the lung parenchyma.

## References

[1] 1. Sezgi C, Cicek M, Sen HS, Kaya H, Taylan M, Abakay A, et al. Pulmonary findings in patients with fascioliasis. Acta Med Mediterr. 2013;29:841-5.

[2] 2. Mas-Coma M, Esteban J, Bargues M. Epidemiology of human fascioliasis: a review and proposed new classification. Bull World Health Organ. 1999;77(4):340-6. PMC2557647 10327713

[3] 3. Sezgin O, Altintas E, Disibeyaz S, Saritas Ü, Sahin B. Hepatobiliary fascioliasis: clinical and radiologic features and endoscopic management. J Clin Gastroenterol. 2004;38(3):285-91. 10.1097/00004836-200403000-00017 15128078

